# Aurintricarboxylic Acid Is a Potent Inhibitor of Influenza A and B Virus Neuraminidases

**DOI:** 10.1371/journal.pone.0008350

**Published:** 2009-12-17

**Authors:** Anwar M. Hashem, Anathea S. Flaman, Aaron Farnsworth, Earl G. Brown, Gary Van Domselaar, Runtao He, Xuguang Li

**Affiliations:** 1 Centre for Biologics Research, Biologics and Genetic Therapies Directorate, HPFB, Health Canada, Ottawa, Ontario, Canada; 2 National Microbiology Laboratory, Public Health Agency of Canada, Winnipeg, Manitoba, Canada; 3 Department of Biochemistry, Microbiology and Immunology, and Emerging Pathogens Research Centre, University of Ottawa, Ottawa, Ontario, Canada; University of British Columbia, Canada

## Abstract

**Background:**

Influenza viruses cause serious infections that can be prevented or treated using vaccines or antiviral agents, respectively. While vaccines are effective, they have a number of limitations, and influenza strains resistant to currently available anti-influenza drugs are increasingly isolated. This necessitates the exploration of novel anti-influenza therapies.

**Methodology/Principal Findings:**

We investigated the potential of aurintricarboxylic acid (ATA), a potent inhibitor of nucleic acid processing enzymes, to protect Madin-Darby canine kidney cells from influenza infection. We found, by neutral red assay, that ATA was protective, and by RT-PCR and ELISA, respectively, confirmed that ATA reduced viral replication and release. Furthermore, while pre-treating cells with ATA failed to inhibit viral replication, pre-incubation of virus with ATA effectively reduced viral titers, suggesting that ATA may elicit its inhibitory effects by directly interacting with the virus. Electron microscopy revealed that ATA induced viral aggregation at the cell surface, prompting us to determine if ATA could inhibit neuraminidase. ATA was found to compromise the activities of virus-derived and recombinant neuraminidase. Moreover, an oseltamivir-resistant H1N1 strain with H274Y was also found to be sensitive to ATA. Finally, we observed additive protective value when infected cells were simultaneously treated with ATA and amantadine hydrochloride, an anti-influenza drug that inhibits M2-ion channels of influenza A virus.

**Conclusions/Significance:**

Collectively, these data suggest that ATA is a potent anti-influenza agent by directly inhibiting the neuraminidase and could be a more effective antiviral compound when used in combination with amantadine hydrochloride.

## Introduction

Influenza viruses cause a highly contagious respiratory tract infection. The frequent mutations of influenza genes, particularly those encoding surface hemagglutinin (HA) and neuraminidase (NA) proteins, allow the virus to evade the host immune system. This gives rise to new infectious strains responsible for annual epidemics associated with significant morbidity and mortality [1], [2]. The recent infections of humans with the highly pathogenic avian H5N1 [3] and swine-origin H1N1 [4] influenza viruses reinforce the notion that the emergence of novel virus strains is unpredictable and capable of threatening the worldwide population [5]. Given the magnitude of a flu pandemic as a threat to the global population, it is crucial to have as many prevention and treatment options as possible.

Vaccines, either inactivated or live attenuated viruses, offer the best protection against influenza infection by inducing neutralizing antibodies against HA and NA antigens of specific influenza strains [6]. However, current vaccines are not ideal because they must be developed and validated annually [6], have lengthy manufacturing and distribution times [7] and short shelf lives [8]. Most importantly, any failure to accurately anticipate the circulating strain results in reduced efficacy or no protection by these vaccines [1], [9]–[12]. These drawbacks, associated with inter-pandemic vaccines, would be drastically exacerbated in the event of a future pandemic [8], [13]. It is therefore crucial to investigate novel therapeutic and preventive anti-influenza agents.

Presently, only two classes of antiviral agents have been developed and approved for prophylaxis and treatment of seasonal influenza infection [5], [14]. The first class blocks the influenza M2 protein, which forms hydrogen ion channels required for the efficient uncoating of incoming viruses [5], [14]. The second class inhibits influenza NA, which is required for efficient release of viral particles from the infected cell [5], [14]. However, resistance to both M2 blockers and NA inhibitors has been reported to be extensive [5], [14]–[16].

Aurintricarboxylic acid (ATA) is a polyaromatic carboxylic acid derivative [17] that inhibits nucleases [18] and nucleic acid processing enzymes [19]. ATA has been shown to inhibit replication of human immunodeficiency [19], [20] and vesicular stomatitis [17] viruses. More recently, we found that ATA could inhibit the severe acute respiratory syndrome-associated coronavirus (SARS-CoV) [21] and vaccinia virus [22]. Here, we report that ATA can substantially inhibit the replication of several strains of influenza A viruses and one-type B virus in tissue cultures with moderate cytotoxicity. We further investigated the combinational effects of ATA and amantadine hydrochloride, an M2 blocker, on the replication of influenza viruses. Finally, we found that ATA inhibits influenza neuraminidase, possibly elucidating its anti-influenza mechanism of action.

## Materials and Methods

### Cell Culture and Viruses

Madin-Darby canine kidney (MDCK) cells (ATCC: CCL-34) were obtained from the American Type Culture Collection (Manassas, VA, USA) and were grown in modified minimum essential medium (modified MEM) containing Earle's balanced salts and supplemented with 2 mM L-glutamine, 1.5 g/l sodium bicarbonate (pH 7.2), 0.1 mM non-essential amino acids, 1.0 mM sodium pyruvate, 10% heat-inactivated fetal bovine serum (FBS), 100 U/ml penicillin and 100 µg/ml streptomycin (Invitrogen, Carlsbad, CA) in a humidified atmosphere of 5% CO_2_. All viruses were amplified and titrated in MDCK cells and stored at −80°C until use. Influenza A strains A/Puerto Rico/8/34 (H1N1) (hereafter referred to as PR8), A/New Caledonia/20/99 (H1N1) (hereafter referred to as NC), and A/New York/55/01 (H3N2) (hereafter referred to as NY) were kindly provided by Dr. Jim Robertson at the National Institute for Biological Standards and Control (Potters Bar, UK). The oseltamivir-resistant influenza virus A/WSN/33 with the substitution of H274Y was made by reverse genetics and provided by Dr. Guy Boivin (Laval University, Quebec City, QC, Canada) and is hereafter referred to as H274Y in this paper. The parental virus influenza A/WSN/33 (H1N1) virus (hereafter referred to as WSN) was obtained from Dr. Earl Brown at University of Ottawa, Ottawa, ON, Canada. Influenza B/Singapore/222/97 virus (hereafter referred to as B) was provided by Dr. Kathryn Wright (Biochemistry, Microbiology and Immunology Department, University of Ottawa, Canada). Confluent MDCK monolayers in 6 or 24-well plates were washed twice with PBS and incubated with viruses at a multiplicity of infection (MOI) of 0.001 in MEM for 2 h at 37°C. After viral adsorption, media was removed, cells were washed twice with PBS and incubated with post-adsorption medium [MEM with 2 mM L-glutamine and Earle's balanced salts supplemented with 100 U/ml penicillin and 100 µg/ml streptomycin, 25 mM HEPES buffer (pH 7.2), 0.1 mM non-essential amino acids, 1.0 mM sodium pyruvate, 2 µg/ml L-1-(tosylamido-2-phenyl) ethyl chloromethyl ketone (TPCK)-treated trypsin (Invitrogen, Carlsbad, CA)]. Plates were incubated for 48 h at 37°C, 5% CO_2_.

### Compounds

Aurintricarboxylic acid (ATA), amantadine hydrochloride (AH), and N-acetyl-2,3-dehydro-2-deoxyneuraminic acid (NAA) were from Sigma (St. Louis, MO, USA). Stock solutions of each drug were prepared immediately before use in dimethyl sulfoxide (DMSO) and filtered using a 0.22 nm filter.

### Neutral Red Assay

Neutral red is a vital dye that is incorporated into the vacuoles of viable cells, can be detected spectrophotometrically and is used to determine cell viability. The in vitro efficacy and toxicity of antiviral compounds have previously been determined by measuring the uptake of neutral red [23]–[25], therefore a neutral red assay kit (Sigma, St. Louis, USA) was used to assess the cytotoxicity and antiviral potential of ATA according to manufacturer's instructions. To determine ATA cytotoxicity, confluent MDCK monolayers in 24-well plates were washed twice with PBS then exposed to ATA in 1 ml post-adsorption medium for 48 h at 37°C, 5% CO_2_. To determine inhibitory potential of ATA alone or in combination with AH, inoculums were aspirated following viral adsorption, cells were washed twice with PBS, then incubated in 1 ml post-adsorption medium containing ATA and/or AH for 48 h at 37°C, 5% CO_2_. DMSO concentration was the same in all treatments. After 48 h, neutral red dye was added to the medium in each well at a concentration of 0.033% and incubated for 2 h at 37°C. The medium was then removed, and cells were fixed by rinsing with 0.1% CaCl_2_ in 0.5% formaldehyde. The incorporated dye was solubilized in 1 ml of 1% acetic acid in 50% ethanol. Cells were incubated at room temperature for 10 min with gentle agitation and the absorbance was measured at 540 nm with a Synergy™ 2 Multi-Mode Microplate Reader. MDCK cells were also evaluated by microscopic examination for cytopathic effect (CPE). Results for ATA cytotoxicity were presented as percentage of absorbance of ATA-treated cells relative to that from untreated controls. ATA and/or AH protection results were expressed as percentage of absorbance of treated or untreated infected cells relative to that from untreated, uninfected controls. The concentration of drug required to inhibit 50% of the CPE induced by PR8 (EC_50_) was determined.

### Reverse Transcription-PCR

Total RNA was extracted from cells 48 h following infection with influenza PR8 and treatment with ATA using an RNeasy mini kit (Qiagen Inc. Valencia, CA). Extracted RNA was treated with DNAse I (Ambion Inc., Streetsville, Ontario, Canada) and single step reverse transcription-PCR was performed using a Titan One Tube RT-PCR kit (Roche Applied Science, 68298 Mannheim, Germany) according to manufacturer's instructions. Influenza PR8 nucleoprotein RNA was amplified from 200 ng RNA in a total reaction volume of 50 µl using the forward and reverse primers 5′-ACTCACATGATGATCTGG-3′ and 5′-CTGCATTGTCTCCGAAGA-3′, respectively. Reverse transcription was performed at 50°C for 30 min. After an initial denaturation at 94°C for 2 min, two amplification steps were performed. The first step consisted of 10 cycles of denaturation at 94°C for 30 sec, annealing at 58°C for 30 sec and elongation at 68°C for 45 sec. The second step consisted of 18 cycles under the same cycling conditions, with 5 sec added to the elongation step in each cycle. After a final extension at 68°C for 7 min, 20 µl of the RT-PCR product was loaded on an 1% agarose gel and visualized by SYBR Green staining and UV exposure.

### Enzyme Linked Immunosorbent Assay (ELISA)

Extracellular influenza A antigens were detected in cell culture supernatants using a commercial ELISA kit (Takara Bio Inc., Otsu, Shiga, Japan). Culture media were removed 48 h after cells were infected with virus and treated with ATA, AH or both. Supernatants were clarified by centrifugation at 5000×g for 5 min. Samples, positive control and standards diluted in diluent/lysis buffer were added to individual wells (100 µl/well), and incubated at 37°C for 1 h with immobilized monoclonal antibody directed against influenza A nucleoprotein (NP), then were washed three times with PBS containing 0.1% Tween-20. Samples were incubated with biotinylated rabbit polyclonal anti-influenza virus antibodies for 1 h at 37°C. Following three washes, streptavidin-o-phenylenediamine dihydrochloride conjugates were added to wells and the plate incubated for 30 min at 37°C. Wells were washed four times and incubated with the substrate solution consisting of hydrogen peroxide and tetramethylbenzidine at room temperature for 15 min. The reaction was stopped by the addition of 1 N H_2_SO_4_ prior to absorbance reading at 450 nm using a Synergy™ 2 Multi-Mode Microplate Reader. Viral abundance was calculated as hemagglutination (HA) units from a standard curve using a positive control with known HA content. One HA unit is equal to the quantity of virus required to completely aggregate erythrocytes in an HA assay (100 µl of 0.25% v/v). The commercial ELISA kit has certain limitations as it employs the anti-nucleoprotein as the capture antibodies coated on the plates and anti-influenza viral proteins (total influenza viral proteins including hemagglutinins) as the detecting antibodies in solution for sandwich ELISA. Because hemagglutinins vary from strain to strain in terms of amino acid sequences or the ratio of viral proteins, one cannot directly compare the HA units between two different strains. Therefore, we present the results as percentage of HA units from treated infected samples relative to that from untreated infected control for the same strain.

### Cell-Associated and Extracellular Virus Yield Reduction Assay

Confluent cells in 6 well plates were washed twice with PBS and inoculated with 1 ml MEM containing PR8 for 2 h at 37 °C. Inoculums were aspirated, cells were washed twice with PBS and treated with 1% DMSO, 100 µg/ml ATA, 100 µg/ml AH or 100 µg/ml NAA for 48 h at 37°C. Cells were scraped off wells and collected by centrifugation at 1600×g for 5 min. Supernatants were transferred to a new tube, then cells were lysed by 2 cycles of freezing and thawing. Supernatants and lysates were subjected to plaque assay as described previously [26] to determine the extracellular and cell-associated virus titer yield upon ATA treatment. Confluent cell monolayers in 6 well plates were incubated with 1 ml of supernatant or lysate at 37°C for 2 h. The inoculum was removed, cells were washed with PBS and overlaid with the maintenance MEM medium containing 0.8% agarose, 0.2% BSA, 2 mM L-glutamine, 1.5 g/l sodium bicarbonate (pH 7.2), 0.1 mM non-essential amino acids, 1.0 mM sodium pyruvate, 100 U/ml penicillin, 100 µg/ml streptomycin and 2 µg/ml TPCK-treated trypsin. After incubation for 3 days at 37°C in a humidified atmosphere of 5% CO_2_, cells were either stained with 0.01% neutral red directly or fixed with 10% formaldehyde, followed by staining with 0.5% crystal violet for plaque counting.

### Direct Inactivation of the Viruses

PR8, NC, NY, WSN, H274Y and influenza B viruses, at a concentration of ∼100 plaque forming units (pfu), were incubated with ATA at 37°C for 30 min. The virus-ATA mixture was transferred to confluent cell monolayers in 6-well plates, incubated at 37°C for 2 h and subjected to plaque assay as described previously [26].

### Electron Microscopy

Confluent cells in 6 well plates were inoculated with PR8 virus for 2 h at 37°C, then treated with DMSO, 100 µg/ml ATA, 100 µg/ml AH in post-adsorption medium for 24 h at 37°C. Cells were scraped off wells and centrifuged at 1600×g for 5 min. Medium was discarded and cells were incubated with ice-cold fixative (2.5% glutaraldehyde in 0.2 M cacodylate buffer, pH 7.4) for 50 min, with gentle agitation. Cells were pelleted by centrifugation at 20,000×g for 2 min at room temperature. Cell pellets were re-suspended in 0.5 ml fixative then rinsed in 0.5 M cacodylate buffer twice for 10 min and post-fixed with 2% osmium tetroxide for 2 h. The fixed cells were washed with water twice for 10 min, dehydrated with increasing concentrations of ethanol from 50 to 100% and embedded in spurr resin. Thin (70–80 nm) sections were cut on an ultramicrotome and counter stained with uranyl acetate and lead citrate. The sections were viewed and photographed on a JEOL 1010 transmission electron microscope.

### Cell-Free Neuraminidase Inhibition Assay

The NA-Star® Influenza Neuraminidase Inhibitor Resistance Detection Kit (Applied Biosystems, Foster City, CA, USA) was used to measure the inhibition of NA activity as described previously [27], [28]. Briefly, serial dilutions of ATA, AH and NAA ranging from 2000 µg/ml to 0.002 µg/ml were prepared in NA-Star assay buffer, i.e. 26 mM MES (2-[N-Morpholino] ethanesulfonic acid), pH 6.0, 4 mM CaCl_2_, such that dilutions were double the final concentration. Twenty-five µl of each drug dilution was combined with 25 µl diluted viruses (PR8, NC, NY, WSN, H274Y and B) or recombinant NA proteins [recombinant N1 protein (influenza A/Beijing/262/95) and N4 protein (influenza A/Gray Teal/Aus/2/79) (Protein Sciences Corporation, Meriden, CT, USA)] in NA-Star assay buffer. Mixtures were incubated at 37°C with gentle agitation for 30 min to allow interaction between drug and virus or recombinant NA protein. Ten µl of NA-Star substrate diluted in assay buffer to a final concentration of 10 µM was added to each mixture in an opaque, solid-white 96-well plate. The reactions were incubated at 37°C for 30 min with gentle agitation, then 60 µl of NA-Star accelerator was added to each well and a chemiluminescent signal was measured at a rate of 1 sec/well in a Synergy™ 2 Multi-Mode Microplate Reader. Curves were constructed by plotting the percentage of NA activity (chemiluminescent signal) in the presence of inhibitors relative to the activity of controls (NA without inhibitors) against the concentration of inhibitors and used to determine the drug concentration required to reduce NA activity to 50% of control NA activity (IC_50_).

### Data Analysis

All data were expressed as means ± standard deviation. NA inhibition assay and IC_50_ calculations were determined using nonlinear curve fit in GraphPad Prism version 5. Statistical analysis was conducted using either one-way or two-way ANOVA when appropriate. To adjust for multiple comparisons, Bonferroni comparison post test was used. P value of <0.05 was regarded as statistically significant.

## Results

### ATA Protects MDCK Cells from Influenza A Strains

**Figure 1:**
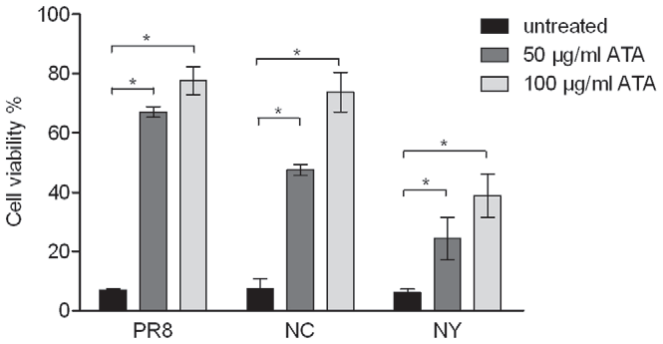
ATA protects MDCK cells from infection with influenza A strains PR8, NY or NC. MDCK cells infected with influenza A viruses (MOI 0.001) were treated with ATA for 48 h. Cell viability was assessed by neutral red assay. The columns represent the means of triplicates and error bars represent standard deviations. * = corrected p-value <0.05.

**Figure 2:**
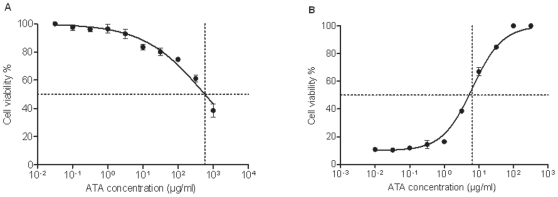
Determination of the selective index of ATA in MDCK cells. (A) Cytotoxicity of ATA in MDCK cells. MDCK cells were treated with ATA for 48 h at the indicated ATA concentrations (µg/ml). Cell viability was determined by neutral red assay by measuring the absorbance at 540 nm. Samples were tested in quadruplicate and showed as means and standard deviations (error bars). (B) Inhibition of influenza A PR8 infection in MDCK cells by ATA is concentration-dependent. MDCK cells were infected with influenza A PR8 virus (MOI 0.001) and treated with ATA at increasing concentrations for 48 h. Cell viability was determined by neutral red assay by measuring the absorbance at 540 nm. Samples were tested in duplicate and showed as means and standard deviations (error bars).

We and others have reported that ATA can inhibit certain RNA viruses; therefore, we postulated that ATA may also inhibit the replication of influenza viruses. To evaluate the ability of ATA to protect MDCK cells from influenza infection, cells inoculated with different influenza A viral strains were incubated in the presence or absence of ATA (50 or 100 µg/ml) for 2 days. Microscopic examination revealed a drastic reduction of influenza-induced CPE following treatment with ATA (data not shown). The viability of MDCK cells infected with influenza A viruses and incubated in the presence or absence of ATA was then assayed by incorporation of neutral red dye. Infected cells treated with ATA had increased neutral red uptake (Fig. 1), confirming that ATA significantly protected MDCK cells from influenza infection (p<0.0001). ATA concentration that caused 50% cytotoxicity (CC_50_) in MDCK cells was determined by assaying neutral red uptake and was found to be 577 µg/ml (Fig. 2A). As shown in Fig. 2B, ATA prevented PR8 infection of MDCK cells in a dose-dependent manner with a minimum effective dose of 6.5 µg/ml required to protect 50% of cells (EC_50_). Therefore, the therapeutic/selective index (SI) which refers to the ratio of the amount of ATA exerting its therapeutic effect to the amount that causes toxicity in MDCK cells is 88.8.

To examine the prophylactic potential of ATA against influenza A infection, MDCK cells were exposed to ATA 24 h prior to viral infection. The cell viability was then assessed by measuring neutral red uptake. We found that pre-exposing MDCK cells to ATA 24 h prior to infection did not protect cells from virus-induced CPE (data not shown). Collectively, these data suggest that ATA is a potent anti-influenza agent with relatively low toxicity in tissue culture, as demonstrated by the SI value of 88.8.

### ATA Inhibits Replication of Influenza A and B Viruses

**Figure 3:**
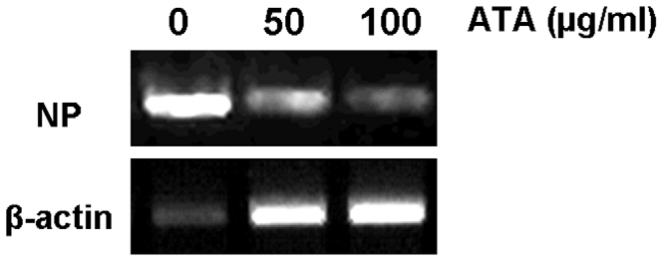
ATA reduces the level of influenza NP RNA detectable in MDCK cells. MDCK cells were infected with influenza A PR8 virus and treated with the indicated concentrations of ATA for 24 h. Total RNA was extracted and reverse-transcription PCR was performed to determine NP and β-actin RNA levels.

To examine whether the protective effect of ATA is due to inhibition of viral replication, the level of influenza nucleoprotein RNA isolated from PR8-infected MDCK cells was determined by reverse-transcription PCR. Cells infected with PR8 had low levels of β -actin RNA (Fig. 3), most likely due to cell death. However, the level of β-actin RNA remained relatively constant upon exposure to increasing ATA concentrations. Treatment with ATA resulted in a substantial reduction in viral NP RNA, compared to untreated cells, suggesting that ATA protects MDCK cells by decreasing viral replication.

**Figure 4:**
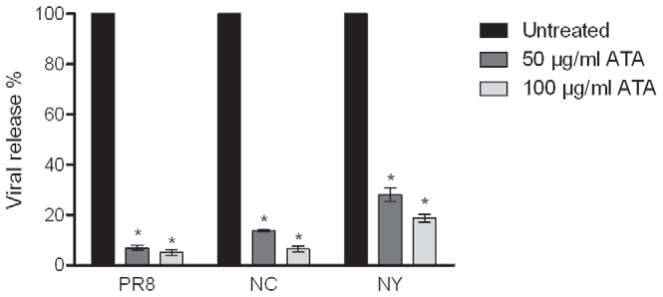
ATA reduces viral release from infected MDCK cells into the media. MDCK cells were inoculated with influenza A viruses (MOI 0.001), then treated with ATA for 48 h. Media were collected and released viruses were quantified by ELISA. The columns represent the means of triplicates and error bars represent standard deviations. * = corrected p-value <0.05.

To determine if ATA treatment also reduces the level of influenza viruses released into the medium, supernatants from ATA-treated infected cell cultures were subjected to the analyses of viral proteins by ELISA. As shown in Fig. 4, treatment of cells with ATA dramatically reduced virus yield (p<0.0001). Doses of 50 µg/ml and 100 µg/ml of ATA reduced the abundance of PR8 by 93% and 95%, NC by 86% and 94%, and NY by only 72% and 81%, respectively. There is no statistically significant difference between the two H1N1 viruses (PR8 and NC), yet the H3N2 virus was slightly more resistant to the ATA treatment (see below for additional discussion).

Since an ELISA measures both infectious and non-infectious particles, we determined whether ATA treatment specifically reduces the abundance of infectious particles. Cells were infected with PR8, then exposed to ATA or other previously established anti-influenza agents, AH, an M2 blocker or NAA, a neuraminidase inhibitor. Virus titres, of both the cell lysate and supernatant fraction, were determined by plaque assay. ATA treatment significantly reduced both the cell-associated and extracellular virus yields when compared to either AH or NAA (Table 1). Moreover, when ATA was included in the maintenance media in a plaque reduction assay with cells infected with either influenza A or B viruses, it reduced both the number and size of plaques (data not shown). Taken together, these data suggest that ATA is a potent inhibitor of influenza A and B viruses.

**Table 1:** Inhibition of Influenza A PR8 virus by ATA, AH and NAA.

	Virus yield (PFU/ml)	
Treatment	Cells	Medium
No treatment	1×10^7^	1×10^7^
DMSO	1×10^7^	1×10^7^
ATA	5×10^4^	1.3×10^4^
AH	4.7×10^5^	3×10^5^
NAA	6.2×10^6^	4.7×10^6^

Confluent MDCK cells in 6 well plates were infected with 0.001 MOI of influenza A PR8 virus and incubated for 48 h with 100 µg/ml of AH, ATA, NAA or DMSO alone. Culture media and cell lysates were collected and viral titers were determined by plaque assay.

### The Inhibition of Influenza Viruses by ATA and AH Is Additive

**Figure 5:**
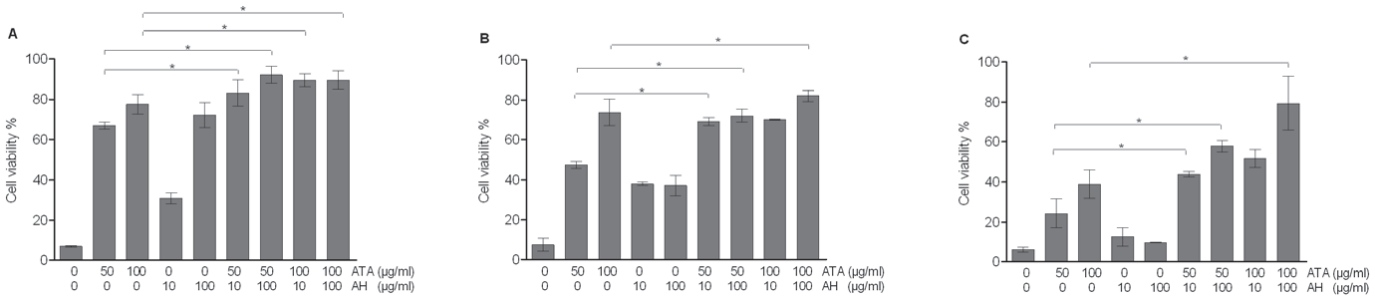
ATA and AH protect MDCK cells from influenza A infection. Uninfected MDCK cells, or MDCK cells infected with influenza A strains were treated with either ATA alone, AH alone, or in combinations at concentrations indicated. (A) MDCK cells infected with influenza A PR8 virus; (B) MDCK cells infected with influenza A NC virus; (C) MDCK cells infected with influenza A NY virus. Protection of cells from infection was determined by NR dye uptake. Cell viability was determined by measuring the absorbance at 540 nm. The columns represent the means of triplicates and error bars represent standard deviations. * = corrected p-value <0.05. Not all statistically significant differences are shown.

**Figure 6:**
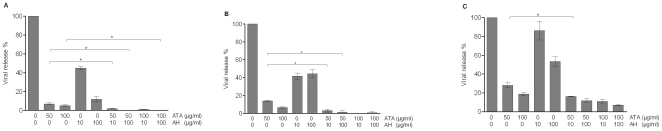
ATA and AH decrease the abundance of viruses released by infected MDCK cells. Viruses in culture supernatants were detected by ELISA 48 h following treatment with ATA, AH or both compounds. (A) MDCK cells infected with influenza A PR8 virus; (B) MDCK cells infected with influenza A NC virus; (C) MDCK cells infected with influenza A NY virus. The columns represent the means of 6 replicates and error bars represent standard deviations. * = corrected p-value <0.05. Not all statistically significant differences are shown.

Upon demonstrating that ATA protects MDCK cells from influenza infection by reducing viral replication and release, we sought to investigate the mechanism underlying the anti-influenza activities of ATA. The protection of influenza-infected MDCK cells should be enhanced by simultaneous treatment with two antivirals acting via different mechanisms. Therefore, MDCK cells infected with influenza were treated concomitantly with ATA and an agent with a known antiviral mechanism. As shown in Fig. 5, the cytopathic effect induced by influenza A viruses in MDCK cells was reduced significantly by concomitant treatment with ATA and AH, compared to either treatment alone. The protective effects of combined ATA and AH treatment were further confirmed by quantitative detection of influenza A viruses present in the culture supernatants by ELISA (Fig. 6). Notably, co-treatment with ATA and AH virtually abolished H1N1 and H3N2 virus production in the media. Since AH effects its anti-influenza properties by targeting the viral M2 protein to prevent efficient uncoating of incoming viral particles, we concluded that inhibition of influenza replication and release by ATA is mediated by a distinct mechanism.

### ATA Inhibits Neuraminidase Activities of Influenza A and B Viruses

**Figure 7:**
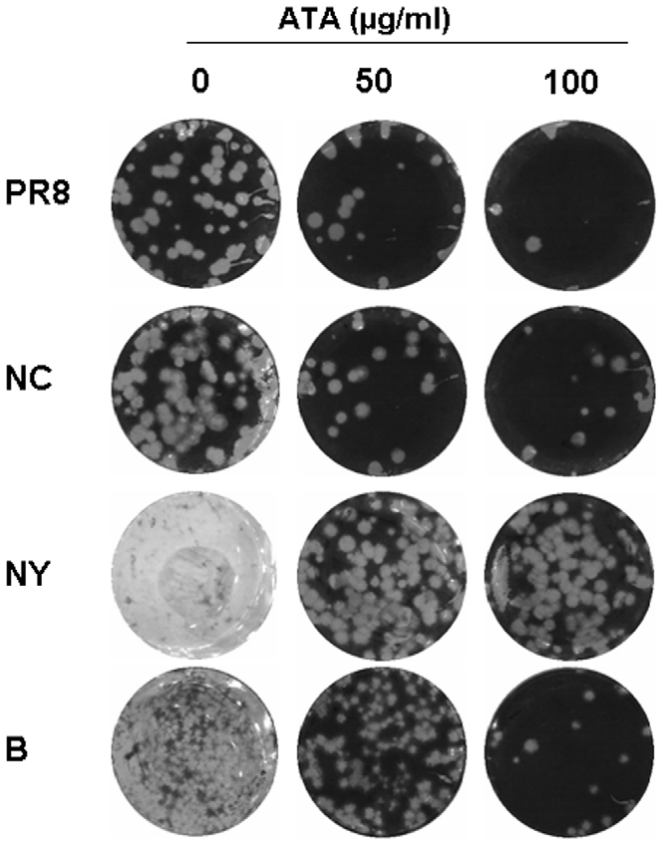
ATA inactivates influenza A and B viruses. Influenza PR8, NC, NY and B viruses were pre-incubated with ATA (0, 50, 100 µg/ml) for 30 min, then the virus-ATA mixture was transferred to confluent cell monolayers in 6-well plates, incubated at 37°C for 2 h and subjected to plaque assay.

To begin to elucidate how ATA protects cells from influenza infection, we investigated whether ATA elicits its inhibitory actions directly on the virus. In previous experiments, the consequences of ATA were examined after viral infection. To determine if ATA inhibits influenza viruses directly, MDCK cells were infected with viruses that had been pre-incubated with ATA, and then subjected to plaque assay. As shown in Fig. 7, pre-incubation of influenza A and B viruses with ATA reduced the number of plaques and protected cells, suggesting that ATA can inactivate viral proteins directly.

**Figure 8:**
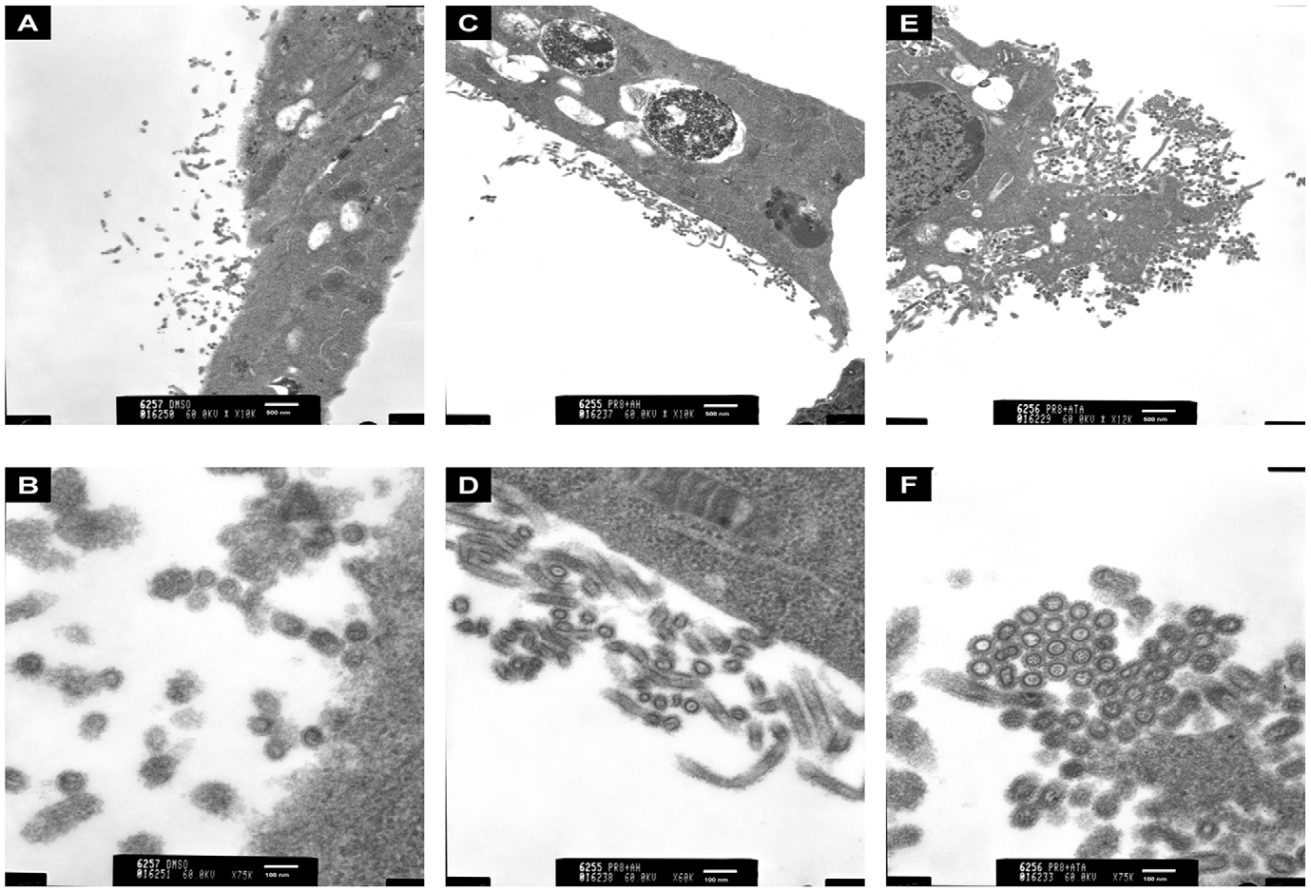
Influenza PR8 virus forms aggregates upon ATA treatment. MDCK cells were infected with influenza A PR8 virus and exposed to DMSO, AH or ATA, then processed for electron microscopy. (A and B) Low and high magnification view, respectively, of MDCK cells infected with influenza A PR8 virus in the presence of DMSO only. (C and D) Low and high magnification view, respectively, of MDCK cells infected with influenza A PR8 virus in the presence of AH. (E and F) Low and high magnification view, respectively, of MDCK cells infected with influenza A PR8 virus in the presence of ATA.

**Figure 9:**
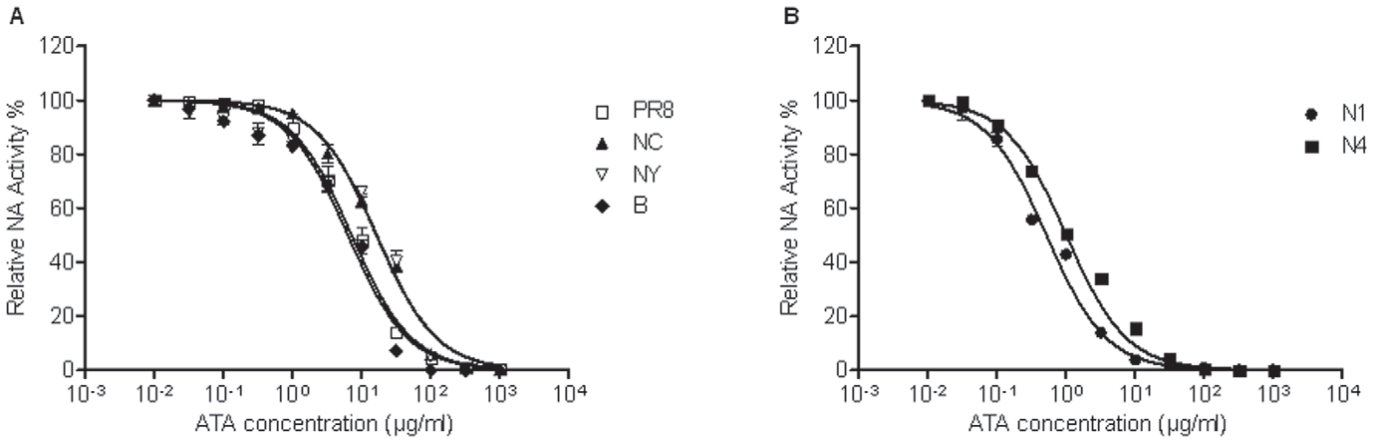
ATA inhibits NA enzymatic activity. ATA inhibits enzymatic activity of NA (A) derived from PR8, NC, NY and B viruses, (B) recombinant N1 and N4 proteins. Viruses or recombinant proteins were incubated with increasing concentrations of ATA and NA enzymatic activity was determined by a chemiluminescent assay. Samples were tested in quadruplicates and presented as means and standard deviations (error bars).

To gain insight into how ATA directly inhibits influenza virus, MDCK cells infected with PR8 and treated with ATA or AH were examined by election microscopy. Compared to untreated PR8-infected cells, or those incubated with AH, ATA treatment was found to induce viral aggregation on the cell surface (Fig. 8). These findings are similar to previous observations by investigators working with NA inhibitors or NA-defective particles [29], [30]. In these studies, NA was believed to prevent aggregation of progeny viruses on the surfaces of infected cells [29], [31], [32]. Therefore, we postulated that ATA elicits viral aggregation by inhibiting the enzymatic activity of NA. To address this question, we tested the effect of ATA on NA activity and compared it to the effect of NAA, a known NA inhibitor [30]. As expected, NAA inhibited NA activity with very low IC_50_ values, while AH showed no inhibition (data not shown). We found ATA inhibited NA activity in a concentration-dependent manner in all viruses, with no marked differences among them. As shown in Fig. 9A, ATA inhibited NA activity from influenza PR8, NC, NY and B viruses with IC_50_s of 7.2 µg/ml, 16.6 µg/ml, 16.4 µg/ml and 6.3 µg/ml, respectively. In addition, ATA significantly inhibited the enzymatic activities of two recombinant NA proteins, N1 and N4 (Fig. 9B) with IC_50_s of 0.52 µg/ml and 1.0 µg/ml, respectively.

### ATA Inhibits Wild-Type and Oseltamivir-Resistant Viruses

**Figure 10:**
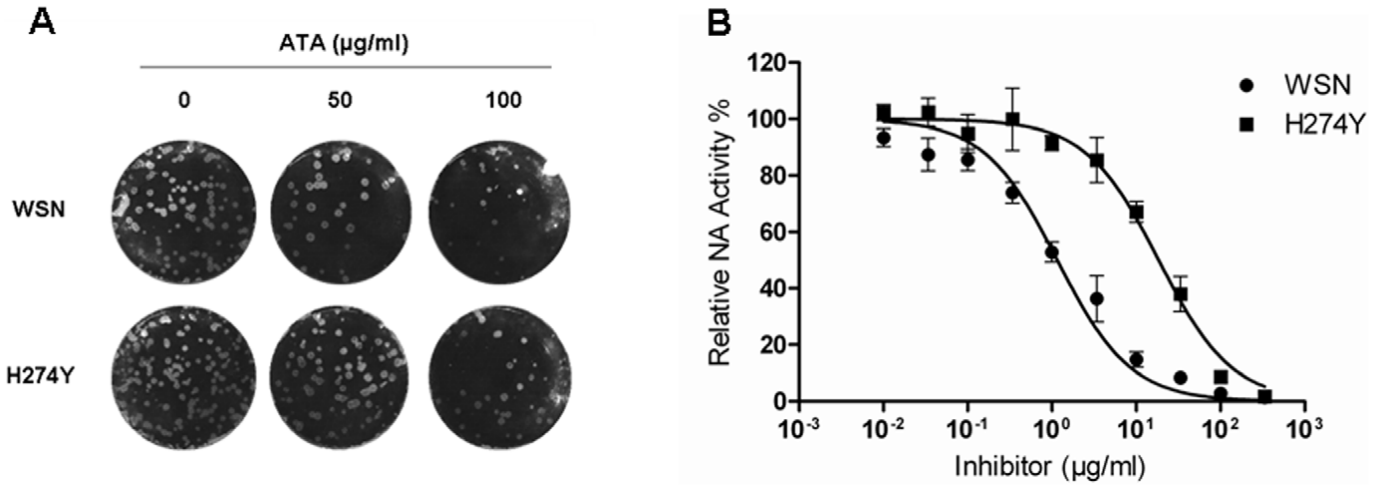
ATA inhibits H274Y oseltamivir-resistant virus. (A) ATA inactivates influenza WSN and H274Y viruses. Influenza WSN and H274Y viruses were pre-incubated with ATA (0, 50, 100 µg/ml) for 30 min, then the virus-ATA mixture was transferred to confluent cell monolayers in 6-well plates, incubated at 37°C for 2 h and subjected to plaque assay. (B) ATA inhibits enzymatic activity of NA derived from WSN and H274Y viruses. WSN (•) and H274Y (▪) viruses were incubated with increasing concentrations of ATA and NA enzymatic activity was determined by a chemiluminescent assay. Samples were tested in quadruplicates and presented as means and standard deviations (error bars).

Next we tested the effect of ATA on a common oseltamivir-resistant viral strain, i.e. WSN (H1N1) virus with the substitution at H274Y, in comparison with the parental wild-type WSN H1N1 virus [33]. As shown in Fig. 10A and 10B, ATA inhibited replication of both WSN and H274Y viruses in a dose-responsive fashion. The IC_50_ values obtained for ATA inhibition of WSN and H274Y viruses were 2 µg/ml and 18 µg/ml, respectively (Fig. 10B). Although the H274Y mutation significantly increased ATA IC_50_ by 9-folds as compared to the wild-type virus, ATA remains a potent inhibitor of both wild-type WSN and H274Y oseltamivir-resistant WSN when compared to the 754-fold increase in IC_50_ of oseltamivir in inhibiting the H274Y mutant [33].

## Discussion

The influenza virus is highly contagious and results in significant morbidity and mortality [2], [34]. While mass vaccination of a susceptible population is the best approach to prevent influenza infections, propensity for mutation and gene reassortment can result in an occasional emergence of novel and unpredicted influenza virus strains. This can give rise to a global influenza pandemic, such as the current triple reassortant swine-origin H1N1 influenza virus [4], [35]. Since considerable time is required to develop and distribute vaccines, novel influenza strains can rapidly spread globally before a vaccine is available for mass immunization. Given the potential for widespread influenza infection, it is crucial to understand and improve treatments for this disease.

Antiviral drugs currently in use (such as the M2 blockers amantadine and rimantidine and NA inhibitors oseltamivir and zanamivir) can reduce the duration of flu symptoms. However, a single mutation can render influenza viruses resistant to antiviral drugs such as M2 blockers or NA inhibitors [36] and drug-resistant influenza strains have been isolated from patients receiving antiviral treatment [15], [16], [37]–[39]. These findings necessitate the exploration of novel anti-influenza agents. We [21], [22] and others [17], [19], [20] have observed that ATA can inhibit a variety of viruses. These observations prompted us to determine the effects of ATA on the replication of influenza virus. In this report, we present data that clearly demonstrates that ATA protects MDCK cells from infection with H1N1 and H3N2 influenza A strains. Specifically, RT-PCR and ELISA analyses indicated that ATA treatment both reduced the intracellular accumulation of viral genomes and the release of influenza viruses into the media. The magnitude of inhibition, as determined by the production of infectious viral particles, was found to be at least 3 logs compared to 1–2 log reduction by either NAA or AH (Table 1).

Although ATA has been reported to have antiviral activity against human immunodeficiency virus [19], [20], vesicular stomatitis virus [17], SARS-CoV [21] and vaccinia virus [22], the mechanism by which ATA inhibits such a diverse group of viruses has remained largely undefined. Various biological activities have been ascribed to ATA. It has been characterized as an inhibitor of nucleic acid processing enzymes [19], nucleases [18], kinase [40] and the JAK-STAT pathway [41], and has been described as an anti-apoptotic factor [40], [42] and insulin-like growth factor [43]. The variety of cellular activities associated with ATA has complicated the elucidation of its antiviral mechanism. While it was previously proposed that ATA inhibits influenza viruses by compromising viral RNA-dependent polymerase [44], we present evidence suggesting that the antiviral activities of ATA could be attributed to the inhibition of the viral neuraminidase. Specifically, we first observed aggregated viral particles on the surface of cells, reminiscent of previous reports by other investigators studying NA inhibitors or NA-defective viruses [29], [30]. This observation led us to conduct a cell-free NA assay, revealing that ATA substantially inhibited the enzymatic activities of both viral and recombinant NA. The difference in the magnitude of inhibition between H1N1 (PR8 and NC) and H3N2 (NY) viruses in infected cultures (Fig.1 and Fig. 4) is not fully understood. Interestingly, no marked differences were observed in the neuraminidase inhibition tests, i.e. the IC_50_ between the three strains (Fig. 9A). The reasons for the discrepancies between tissue culture studies and in vitro enzymatic studies are still unclear. It is plausible that the in vitro enzymatic studies may not accurately reflect the events in live cell cultures. Moreover, the IC_50_ in the oseltamivir-resistant strain (H274Y) only increased by 9-fold (Fig. 10B). We think that ATA is still a potent inhibitor for the oseltamivir-resistant strain when compared with oseltamivir in inhibiting the H274Y mutant, for which an over 700- fold increase of IC_50_ is needed [33]. It would be interesting to investigate potential ATA resistant mutants with respect to NA amino acids mutations.

During the preparation of this manuscript, we noted that Hung et al. [45] also reported that ATA protects cells from a variety of virus strains in vitro and can inhibit NA in cell-free assays. In our studies, the IC_50_ and selective index are higher than those reported by Hung et al. [45], but we suspect that such discrepancies might be due to differences in experimental conditions. While our two groups have demonstrated that viral NA activity is compromised by ATA, it is possible that ATA also targets other viral or cellular proteins to exert its anti-influenza activity. Additional studies are required to answer this question.

Since inhibition of influenza by ATA and AH is mediated by two distinct mechanisms, it is not surprising that we observed additive effects upon simultaneous treatment with both compounds. Recently the Advisory Committee on Immunization Practices (ACIP) recommended against the use of amantadine or rimantidine to treat influenza infection [34] due to increasing evolution of M2 blocker-resistant influenza strains [14], [15]. Although influenza strains resistant to NA inhibitors are less prevalent [5], resistance to oseltamivir has also been reported [15], [46]. This suggests that the use of a single class of antiviral may have limited protective value and future influenza treatment strategies will likely include combinations of medications. Notably, combined used of both M2 blockers and NA inhibitors does provide additive protection against influenza infection compared to either treatment alone [26], [47]. Mice infected with 50% lethal doses of either amantadine-sensitive or amantadine-resistant H5N1 influenza, were more protected by co-treatment with amantadine and oseltamivir than those treated with one drug only [47]. We found that simultaneous treatment with ATA and AH significantly protected MDCK cells from influenza and dramatically reduced the abundance of influenza particles released in the medium.

The toxicity of ATA will need to be evaluated further in animals. In this study, we showed that ATA is associated with relatively low toxicity in tissue cultures, with the SI being around 88.8. Although in vivo toxicity studies of ATA are rather limited, previous research in hamsters has shown that infusion of ATA was well tolerated in a dose of up to 1 mg/kg/hour for 2 weeks [48]. Also, Jan Balzarini et al. [20] have found that a single ATA dose of 340 mg/kg in NMRI mice was associated with LD_50_ and that mice had a median life span of 18 days upon intra-peritoneal administration of 31 mg/kg/day. Intra-tracheal inhalation showed that single doses of ATA as high as 4 mg/kg were tolerated well in mice [49], [50]. However, the therapeutic and toxic doses would have to be determined in animal studies, which are currently under investigation in our laboratory.

In short, ATA is an NA inhibitor that may prove to be a valuable inclusion to the current arsenal of anti-influenza agents. The data presented here provide compelling evidence to further study the anti-influenza potential of ATA in animal models.
