# Correction: Aurintricarboxylic Acid Is a Potent Inhibitor of Influenza A and B Virus Neuraminidases

**DOI:** 10.1371/annotation/eb17187c-4618-4558-89e4-322d4acc9b04

**Published:** 2013-06-24

**Authors:** Anwar M. Hashem, Anathea S. Flaman, Aaron Farnsworth, Earl G. Brown, Gary Van Domselaar, Runtao He, Xuguang Li

There is an affiliation missing for author Anwar M. Hashem. The following is the missing affiliation:

Department of Microbiology, Faculty of Medicine, King Abdulaziz University, Jeddah, Saudi Arabia 

